# Genetic Deletion of RHAMM Alleviates Hepatic Oxidative Stress, Reversing Thyroid Stimulating Hormone Elevation in Male Obese Mice

**DOI:** 10.3390/cells14181448

**Published:** 2025-09-16

**Authors:** Tianzhen Wang, Helin Sun, Ayman K. Banah, Xiong Weng, Sharadha Dayalan Naidu, Dot Kisielewski, Abel Ang, John D. Hayes, Albena T. Dinkova-Kostova, Li Kang

**Affiliations:** 1 Division of Diabetes, Endocrinology, and Reproductive Biology, School of Medicine, University of Dundee, Dundee DD1 9SY, UK; tianzhen0310@outlook.com (T.W.); 2646663@dundee.ac.uk (H.S.); akbanah@uqu.edu.sa (A.K.B.); xweng@ed.ac.uk (X.W.); 2 Breast and Thyroid Surgery, Changchun University of Chinese Medicine, Changchun 130117, China; 3 Division of Cancer Research, School of Medicine, University of Dundee, Dundee DD1 4HN, UK; s.z.dayalannaidu@dundee.ac.uk (S.D.N.); d.j.kisielewski@dundee.ac.uk (D.K.); aang001@dundee.ac.uk (A.A.); j.d.hayes@dundee.ac.uk (J.D.H.); a.dinkovakostova@dundee.ac.uk (A.T.D.-K.)

**Keywords:** RHAMM, obesity, thyroid dysfunction, oxidative stress, Nrf2

## Abstract

**Objective**: Obesity induces hypothyroidism with unknown mechanisms. This study investigates the role of (Receptor for Hyaluronan-Mediated Motility (RHAMM) in obesity-associated thyroid dysfunction, focusing on hepatic oxidative stress. **Methods**: Global RHAMM-deficient mice and their wildtype littermate controls were fed a normal chow diet or high-fat diet (HFD) for 16 weeks. Thyroid function was evaluated by measuring plasma thyroid-stimulating hormone (TSH) levels. The hepatic oxidative response was assessed by measuring signaling pathways associated with nuclear factor erythroid 2-related factor 2 (Nrf2) activity. **Results**: HFD feeding increased plasma TSH levels in male mice but not in female mice. RHAMM deletion in male mice mitigated HFD-induced TSH elevation, which was associated with enhanced hepatic antioxidant defenses and reduced inflammation. This was evidenced by elevated expression of the Nrf2 target gene NAD(P)H: quinone oxidoreductase 1 (Nqo1), reduced protein carbonylation and nitration levels, and reduced expression of the pro-inflammatory cytokines IL-1β and TNF-α in livers of male RHAMM-deficient mice. Mechanistically, RHAMM deletion decreased AKT/ERK signaling, increased GSK3 signaling, increased CD44 protein expression, and increased Nqo1 levels in the liver. **Conclusions**: RHAMM promotes obesity-induced thyroid dysfunction by regulating oxidative stress and inflammation in male mice. Targeting RHAMM may provide a novel therapeutic strategy for mitigating obesity-related endocrine and metabolic disorders.

## 1. Introduction

Obesity is a global epidemic, significantly contributing to many chronic diseases, including Type 2 diabetes, heart failure, and cancer. Among its complications, thyroid dysfunction has emerged as a critical concern due to its essential role in maintaining energy homeostasis [1]. Elevated thyroid-stimulating hormone (TSH) levels are consistently observed in obese individuals [2]. However, the underlying mechanisms remain poorly understood [3].

Thyroid hormones regulate energy expenditure and hepatic metabolism via the hypothalamus–pituitary–thyroid (HPT) axis, influencing insulin sensitivity and suppressing hepatic gluconeogenesis [4,5]. Subclinical hypothyroidism, characterized by elevated TSH, is associated with metabolic dysfunction-associated steatotic liver disease (MASLD), where higher TSH levels correlate with increased steatosis severity [6]. Vice versa, the liver plays a crucial role in thyroid hormone metabolism by converting thyroxine (T4) to its biologically active form, triiodothyronine (T3) [7]. The thyroid gland, reliant on reactive oxygen species (ROS) for hormone synthesis, is particularly susceptible to oxidative damage [8]. Moreover, obesity-induced oxidative stress disrupts hepatic function, impairing thyroid hormone metabolism and the liver–thyroid axis [9]. Therefore, protecting the liver from oxidative damage may restore thyroid hormone metabolism and function in obesity.

The extracellular matrix (ECM) is a dynamic network of proteins, proteoglycans, polysaccharides, and biologically active factors that provide structural support and information about the status of the extracellular environment to cells [10]. Emerging evidence highlights the role of ECM remodeling in obesity-related metabolic dysfunction, including insulin resistance [11]. Hyaluronan (HA), a major ECM glycosaminoglycan, and its cell membrane receptors, CD44 and RHAMM (encoded by *Hmmr*), contribute to obesity-associated insulin resistance and cardio-renal dysfunction by promoting inflammation and fibrosis [12,13,14,15]. ROS-induced depolymerization of HA generates fragments that sustain chronic inflammation and fibrosis, establishing a feedback loop of oxidative damage and inflammation [16].

Nrf2, a key regulator of the cellular antioxidant responses, mitigates oxidative stress by inducing glutathione- and thioredoxin-antioxidant enzymes, as well as detoxification enzymes such as Nqo1 [17,18]. Previous study suggests HA signaling via CD44 and RHAMM regulates Nrf2 expression through AKT phosphorylation, linking HA remodeling to redox homeostasis [19]. Nevertheless, the specific role of RHAMM in metabolic regulation, hepatic oxidative stress, and thyroid dysfunction in obesity remains unknown. This study tested the hypothesis that RHAMM deletion enhances hepatic antioxidant defenses by increasing Nrf2 activity, alleviating obesity-induced hepatic oxidative stress and restoring thyroid hormone homeostasis. Our results uncovered a novel role of RHAMM in obesity-induced thyroid dysfunction.

## 2. Materials and Methods

### 2.1. Animal Model

All animal procedures were conducted in compliance with UK Home Office regulation and followed the ARRIVE guidelines, with oversight from the Welfare and Ethical Use of Animals Committee at the University of Dundee. All animals were housed and maintained in a controlled environment with a temperature of 22 ± 1 °C and regulated humidity under a 12 h light/dark cycle. Animals had free access to food and tap water.

Global RHAMM-deficient mice were generously provided by Prof. Eva Turley from Western University (London, ON, Canada). Male and female RHAMM knockout mice (*Hmmr*^−/−^) and their wildtype littermate controls (*Hmmr*^+/+^) were obtained by breeding heterozygous mice [20]. After weaning, all mice were maintained on a regular chow diet (LabDiet 5001, St. Louis, MO, USA). From the age of 6 weeks, one group of *Hmmr*^+/+^ and *Hmmr*^−/−^ mice continued this chow diet for an additional 16 weeks (CHOW), while another group of *Hmmr*^+/+^ and *Hmmr*^−/−^ mice were switched to a 60% high-fat diet (HFD) (#824054, SDS) for 16 weeks. At the end of the feeding period (22 weeks of age), mice were sacrificed under anesthesia, and plasma and liver tissues were harvested for further analyses. Plasma samples were stored at −20 °C and tissue samples were stored at −80 °C. Samples were collected from both male and female mice. However, due to the lack of a dietary response in thyroid-stimulating hormone (TSH) level and its high variability in the female wildtype mice (see Results), only plasma and tissue samples from the male *Hmmr*^−/−^ mice were studied to assess the role of RHAMM in obesity-associated thyroid dysfunction.

Global Nrf2 knockout (Nrf2 KO) and Keap1 knockdown (Keap1 KD, i.e., *Keap1^FA/FA^* hypomorph) mice and their wildtype control mice (WT) [21,22,23,24] were maintained on a regular chow diet (LabDiet 5001, St. Louis, MO, USA) before being humanely sacrificed for plasma and liver tissue collection at the age of 8–17 weeks. Plasma samples were stored at −20 °C and tissue samples were stored at −80 °C before further biochemical analyses. Male mice were used for reasons described above.

### 2.2. RNA Extraction and Real-Time PCR

Total RNA was isolated from liver tissues of mice using the TRIzol reagent (Ambion (Austin, TX, USA), #20130301) and subsequently reverse transcribed into complementary DNA (cDNA) using the cDNA Reverse Transcription Kit (Thermo Fisher (Waltham, MA, USA), #18064014). Quantitative PCR (qPCR) was performed on a QuantStudio 3 Real-Time PCR System (Applied Biosystems, Foster City, CA, USA), using the following Taqman probes: Nrf2 (Mm00477784_m1), Nqo1 (Mm01253561_m1), Hmox1 (Mm00516005_m1), Gclm (Mm01324400_m1), IL-6 (Mm00446190_m1), IL-10 (Mm00439614_m1), IL-1β (Mm00434228_m1), TNF-α (Mm00443260_g1), CD44 (Mm01277161_m1), Hmmr (Mm00469183_m1), and 18S (Mm03928990_g1). All reactions were conducted in triplicates. Relative gene expression levels were determined using the 2^−ΔΔCt^ method, with normalization to the expression of 18S.

### 2.3. Protein Extraction and Western Blotting

Proteins in livers of mice were extracted by homogenization in lysis buffer (50 mM Tris, pH 6.8, 2% SDS, 10% glycine, and 0.1% β-mercaptoethanol) supplemented with protease and phosphatase inhibitors (Thermo Fisher, #78430, #A32961). Proteins (15–30 µg) were separated on 10% SDS-PAGE gels and transferred to nitrocellulose membranes. Protein levels were measured by immunoblotting using primary antibodies (1:1000) specific to Nrf2, Nqo1, Nitrotyrosine, p-AKT(S473), AKT, p-ERK, ERK, and p-GSK3, and GSK3 (Cell Signaling (Danvers, MA, USA), #20733S, #62262, #0409, #9271, #9272, #4370, #4695, #8566, and #5676). GAPDH (Cell Signaling, #5174) or β-actin (Cell Signaling, #4970) served as loading controls.

### 2.4. Plasma Thyroid-Stimulating Hormone (TSH)

Plasma TSH levels were measured using an enzyme-linked immunosorbent assay (ELISA) kit (Thermo Fisher, #EEL110). Absorbance was measured at 450 nm using a microplate reader, and TSH concentrations were calculated based on a standard curve generated from known TSH concentrations. All measurements were performed in triplicates.

### 2.5. Quantification of Liver Triglycerides

Approximately 100 mg of liver tissue was homogenized in 3 M potassium hydroxide (KOH) dissolved in 65% ethanol to quantify liver triglyceride content using the glycerol phosphate oxidase-based triglyceride reagent (Pointe Scientific (Canton, MI, USA), #T7532-500). Absorbance of the colorimetric reaction was acquired at 500 nm using a Multiskan microplate reader (Thermo Fisher).

### 2.6. Protein Oxidation Levels Measurement

Oxidation of proteins in liver tissue was assessed using the OxyBlot™ Protein Oxidation Detection Kit (Merck Millipore (Darmstadt, Germany), #S7150). Livers were homogenized in lysis buffer (50 mM Tris pH 6.8, 2% SDS, 10% glycine) and protein concentrations were determined using the BCA assay (Thermo Fisher, #23228 and #23224). For each sample, 20 µg of protein was derivatized with 2,4-dinitrophenylhydrazine (DNPH) to detect carbonyl groups. Protein carbonylation was then detected by immunoblotting using an anti-DNPH antibody (1:150), followed by an HRP-conjugated secondary antibody (1:300). Oxidized proteins were visualized by enhanced chemiluminescence.

### 2.7. Malondialdehyde (MDA) Level Measurement

Liver MDA levels were quantified using the thiobarbituric acid reactive substances (TBARS) assay kit (Cayman Chemical (Ann Arbor, MI, USA), #10009055). Liver tissues (25 mg) were homogenized in RIPA buffer (Cayman Chemical, #10010263) and centrifuged at 1600× *g* for 10 min at 4 °C. The supernatant was collected for analysis. For the TBARS assay, 100 μL of supernatant was mixed with thiobarbituric acid (TBA) reagent and incubated at 95 °C for 60 min. After cooling, the samples were subjected to centrifugation, and the absorbance of the MDA-TBA adduct was measured at 535 nm in the supernatant. MDA concentrations were calculated using a standard curve and expressed as nanomoles of MDA per gram of tissue weight (nmol/g tissue).

### 2.8. Statistical Analysis

All data were assessed for normal distribution and subjected to statistical analysis using either one-way or two-way ANOVA with Tukey’s multiple comparisons test. Results were reported as mean ± SEM, with significance levels denoted as * *p* < 0.05, ** *p* < 0.01, *** *p* < 0.005, and **** *p* < 0.001. All graphical representations of the data were created using Prism10 (GraphPad, San Diego, CA, USA).

## 3. Results

### 3.1. Global Hmmr Gene Deletion Mitigates Obesity-Induced Increases in Plasma TSH Concentrations in Male Mice

**Figure 1:**
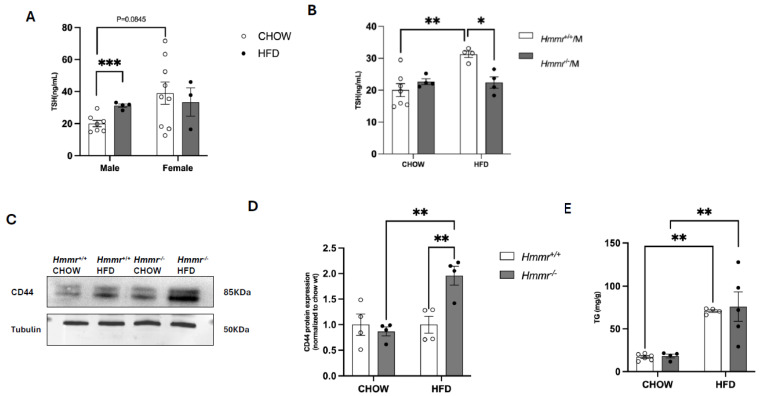
*Global Hmmr* gene deletion attenuates obesity-induced elevation of TSH in male mice. (**A**) Plasma TSH levels were measured in male and female mice fed a high-fat diet (HFD) or a standard chow diet (CHOW). (**B**) TSH levels were measured in male *Hmmr*^+/+^ and *Hmmr^−/−^* mice under both dietary conditions. The same samples were used for male *Hmmr*^+/+^ mice in both panels (**A**,**B**). (**C**,**D**) Western blot analysis and quantification of CD44 protein expression levels in *Hmmr*^+/+^ and *Hmmr*^−/−^ mice under different dietary conditions. Samples from heterozygous *Hmmr*^+/−^ mice were included for reference. (**E**) Hepatic triglyceride (TG) levels were measured in *Hmmr*^+/+^ and *Hmmr*^−/−^ mice under different dietary conditions. Sample sizes were presented as the number of data points. Data were analyzed using two-way ANOVA with Tukey’s multiple comparisons. *p* < 0.05 (*), *p* < 0.01 (**), and *p* < 0.001 (***).

HFD feeding caused a significant increase in plasma TSH concentrations in male but not in female mice (Figure 1A). Although not statistically significant (*p* = 0.08), female mice fed a normal chow diet tended to have higher plasma TSH levels than their male counterparts (Figure 1A). Because of the absence of dietary effect and the high variability in TSH levels in female mice, male mice were studied to investigate the role of RHAMM in obesity-induced thyroid dysfunction. Global deletion of the RHAMM-encoding *Hmmr* gene (*Hmmr*^−/−^) in male mice did not affect body weight gain [15] or body composition but abolished the HFD-induced increases in plasma TSH levels, with significantly lower TSH levels in HFD-fed *Hmmr*^−/−^ mice relative to HFD-fed wildtype littermates (*Hmmr*^+/+^) (Figure 1B). These results suggest that RHAMM regulates thyroid function in obesity.

The liver plays a central role in regulating thyroid function through various mechanisms, particularly in the metabolism, activation, and clearance of thyroid hormones. To investigate how RHAMM regulates thyroid function, hepatic cellular signaling pathways were studied in *Hmmr*^−/−^ mice. CD44 and RHAMM are co-receptors for hyaluronan. Genetic deletion of RHAMM caused a compensatory increase in CD44 protein expression in HFD-fed *Hmmr*^−/−^ mice compared to HFD-fed *Hmmr*^+/+^ mice but with no effects in the chow-fed mice (Figure 1C,D). While HFD feeding markedly elevated hepatic triglyceride levels in mice of both genotypes, deletion of RHAMM did not influence diet-induced triglyceride accumulation in the liver (Figure 1E).

### 3.2. RHAMM Deletion Suppresses AKT/ERK and Activates GSK3 Signaling Pathways and Induces Nqo1 in Obese Mice

**Figure 2:**
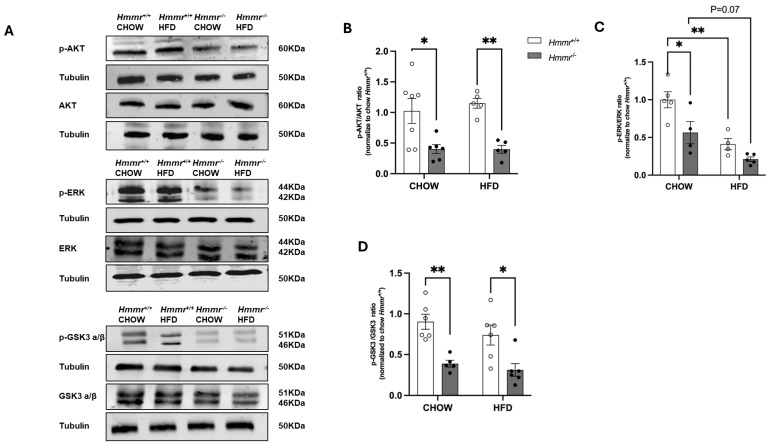
*Global Hmmr* gene deletion modulates AKT, ERK, and GSK3 signaling pathways in the liver under chow and HFD conditions. (**A**) Representative Western blot images show phosphorylated AKT (p-AKT), total AKT, phosphorylated ERK1/2 (p-ERK1/2), total ERK1/2, phosphorylated GSK3α/β (p-GSK3α/β), and total GSK3α/β in liver samples from *Hmmr^+/+^* and *Hmmr^−/−^* mice. (**B**–**D**) Protein levels were normalized to GAPDH. Sample sizes were presented as the number of data points. Data were analyzed using two-way ANOVA with Tukey’s multiple comparisons. *p* < 0.05 (*) and *p* < 0.01 (**).

RHAMM can form a complex with ERK1/2 and activate the protein kinase in breast cancer cells [25]. Here we observed that the levels of phosphorylation of AKT, ERK, and GSK3 α/β were significantly lower in the livers of *Hmmr*^−/−^ mice compared to those of their *Hmmr*^+/+^ counterparts under normal chow diet (Figure 2). HFD feeding decreased phosphorylation of ERK in the livers of *Hmmr*^+/+^ mice (Figure 2A,C). Furthermore, when placed on an HFD, phosphorylation of AKT and GSK3 α/β was lower in the livers of HFD-fed *Hmmr*^−/−^ mice relative to those of *Hmmr*^+/+^ mice (Figure 2A,B,D).

**Figure 3:**
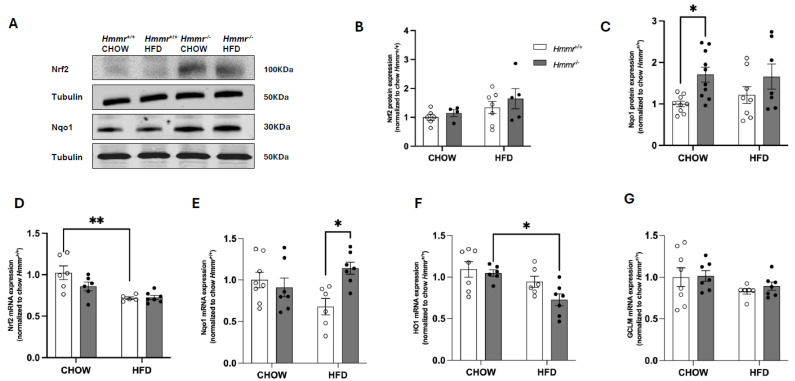
*Global Hmmr* gene deletion increases Nrf2 target gene Nqo1 expression in liver tissues. (**A**–**C**) Representative Western blot images and quantification of Nrf2 and Nqo1 protein expression in liver from *Hmmr^+/+^* and *Hmmr^−/−^* mice. β-actin or GAPDH served as loading controls. Protein levels were normalized to the *Hmmr^+/+^* chow group. (**D**–**G**) Relative mRNA expression levels of Nrf2, Nqo1, HO1, and GCLM, normalized to *Hmmr^+/+^* CHOW, assessed by qRT-PCR. Sample sizes were presented as the number of data points. Data were analyzed using two-way ANOVA with Tukey’s multiple comparisons. *p* < 0.05 (*) and *p* < 0.01 (**).

GSK3 mediates the phosphorylation and subsequent β-TrCP-mediated proteasomal degradation of transcription factor Nrf2, which plays a critical role in the cellular defense against oxidative stress [26]. Therefore, we next measured Nrf2-mediated signaling pathways. Steady-state protein levels of Nrf2 were not affected either by diet or genotype, though marked interindividual differences were apparent (Figure 3A,B). Intriguingly, the protein levels of Nqo1, encoded by a gene that is a well-established target of Nrf2, were significantly higher in chow-fed *Hmmr*^−/−^ mice than chow-fed *Hmmr*^+/+^ mice, without differences in HFD-fed mice (Figure 3A,C). Furthermore, the mRNA levels for *Nfe2l2*, the gene encoding Nrf2, were decreased by HFD feeding in *Hmmr*^+/+^ mice but were unaffected by genotype regardless of diet (Figure 3D). The mRNA levels for Nqo1 were significantly upregulated in *Hmmr*^−/−^ mice fed HFD (Figure 3E). The mRNA for HO1 (encoded by *Hmox1*) was reduced considerably in *Hmmr*^−/−^ mice on HFD relative to the chow diet (Figure 3F), whereas Gclm mRNA showed no differences between genotypes or between diets (Figure 3G).

### 3.3. Global Hmmr Gene Deletion Decreases Hepatic Oxidative Stress and Inflammation in Obese Mice

**Figure 4:**
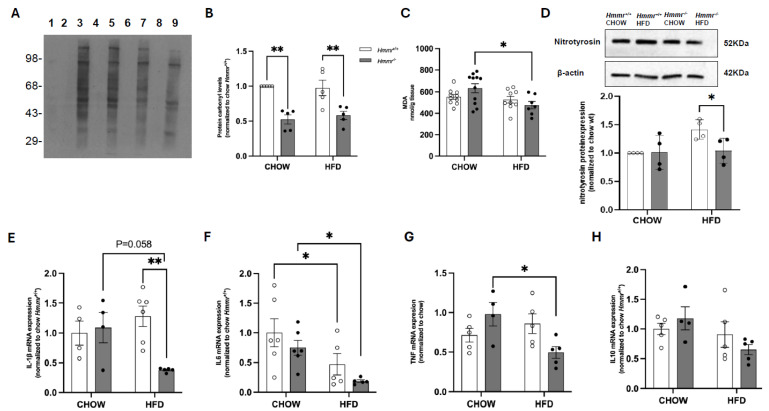
Genetic *hmmr* deletion ameliorates hepatic oxidative stress and inflammation. (**A**) A representative OxyBlot image shows the levels of oxidized proteins. Lanes: 1, molecular weight standard; 2, 4, 6, 8, negative controls; 3, CHOW-*Hmmr^+/+^*; 5, HFD-*Hmmr^+/+^*; 7, CHOW-*Hmmr^−/−^*; 9, HFD-*Hmmr^−/−^*. The graph shows a densitometric analysis of lanes 3, 5, 7, and 9 normalized to CHOW-*Hmmr^+/+^*. (**B**) Quantification of protein carbonyl levels in liver tissue. (**C**) Measurement of malondialdehyde (MDA) levels as an indicator of lipid peroxidation in liver tissues. (**D**) Representative Western blot images and quantification of nitrotyrosine protein expression in liver of *Hmmr^+/+^* and *Hmmr^−/−^* mice. β-actin served as loading control. Protein levels were normalized to the *Hmmr^+/+^* chow group. (**E**–**H**) Relative mRNA expression of IL-1β, IL-6, TNF-α, and IL-10, normalized to CHOW-*Hmmr^+/+^*, analyzed by qRT-PCR. Sample sizes were presented as the number of data points. Data were analyzed using two-way ANOVA with Tukey’s multiple comparisons. *p* < 0.05 (*) and *p* < 0.01 (**).

Changes in the mRNA and protein expression of Nqo1 by RHAMM deletion suggest that RHAMM might modulate hepatic redox homeostasis. Indeed, Western blot analysis showed a marked reduction in protein carbonylation, a marker of oxidative protein damage, in the livers of *Hmmr*^−/−^ mice under both chow and HFD conditions (Figure 4A,B). However, malondialdehyde (MDA) levels, a marker of lipid peroxidation, did not differ between *Hmmr*^+/+^ and *Hmmr*^−/−^ mice regardless of diet, although MDA levels were significantly decreased by HFD feeding in *Hmmr*^−/−^ mice (Figure 4C). To further confirm the antioxidative effects, we examined protein levels of nitrotyrosine as a marker of protein nitration and oxidative stress. Nitrotyrosine levels were significantly decreased in the livers of HFD-fed *Hmmr*^−/−^ mice relative to those in HFD-fed *Hmmr*^+/+^ mice (Figure 4D). We next assessed the impact of RHAMM deletion on inflammation. Intriguingly, qRT-PCR analysis revealed that HFD caused a reduction in IL-6 mRNA levels, without affecting mRNA levels of IL-1β, TNFα, or IL-10 in *Hmmr*^+/+^ mice (Figure 4E–H). Similarly, HFD feeding decreased or tended to decrease mRNA levels of IL-1β, IL-6, and TNFα in *Hmmr*^−/−^ mice, without affecting IL-10. However, IL-1β mRNA expression was significantly downregulated in HFD-fed *Hmmr*^−/−^ mice compared to HFD-fed *Hmmr*^+/+^ mice, indicative of an anti-inflammatory response (Figure 4E). These results suggest that global *Hmmr* deletion might reduce hepatic oxidative damage and inflammation, particularly under HFD conditions.

### 3.4. Nrf2 Suppresses CD44 and RHAMM Expression and Regulates Thyroid Function in Mice

**Figure 5:**
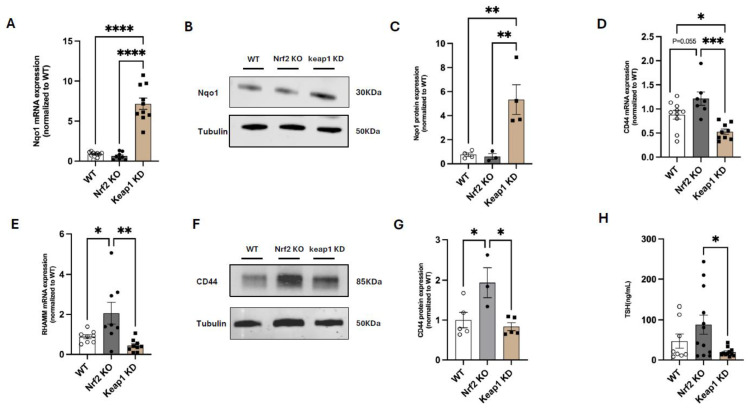
Nrf2 activation decreases CD44 and RHAMM expression and suppresses plasma TSH concentrations in mice. mRNA expression levels of Nqo1 (**A**), CD44 (**D**), and RHAMM (**E**) were measured in the liver tissues of WT, Nrf2 KO, and Keap1 KD mice. Representative Western blot images (**B**,**F**) and quantification of Nqo1 (**C**) and CD44 (**G**) protein expression in liver from WT, Nrf2 KO, and Keap1 KD mice. β-actin or GAPDH served as loading controls. (**H**) Plasma TSH concentrations. Sample sizes were presented as the number of data points. Data were analyzed using one-way ANOVA. Statistical significance is indicated as follows: *p* < 0.05 (*), *p* < 0.01 (**), *p* < 0.001 (***), and *p* < 0.0001 (****).

To explore the potential role of Nrf2 as a mediator of thyroidal regulation by RHAMM, we obtained liver samples from male global Nrf2-knockout (Nrf2 KO) mice and male global Keap1-knockdown (Keap1 KD) mice, respectively. As expected, the mRNA and protein levels of Nqo1 were markedly increased in Keap1 KD mice when compared with wildtype (WT) controls or Nrf2 KO mice (Figure 5A–C). Importantly, Nrf2 KO mice showed a trend of upregulation or marked upregulation of CD44 and RHAMM mRNA relative to WT mice. Conversely, Keap1 KD mice, where Nrf2 activity is constitutively upregulated, displayed significantly reduced gene expression of both RHAMM and CD44, when compared to Nrf2 KO mice (Figure 5D,E). Consistent with the differences in gene expression, CD44 protein expression was higher in Nrf2 KO mice relative to WT controls and Keap1 KD mice (Figure 5F,G). These data are in agreement with the negative correlation between Nrf2 and CD44 described in human melanoma samples [27]. Intriguingly, TSH levels were significantly lower in Keap1 KD mice when compared with those in Nrf2 KO mice (Figure 5H). These results suggest that, while RHAMM affects Nrf2 activation, Nrf2 modulates CD44 and RHAMM expression, which may provide a feedback regulation.

## 4. Discussion

**Figure 6:**
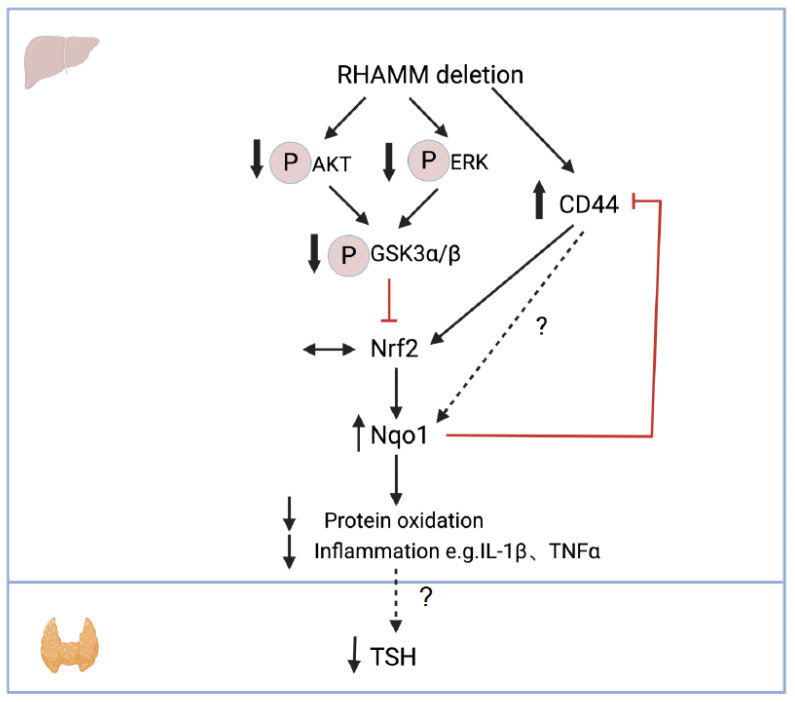
A working model by which RHAMM regulates thyroid function via modulating Nqo1-mediated hepatic oxidative stress and inflammation. Genetic deletion of RHAMM decreases AKT/ERK signaling, increases GSK3 activity, and increases CD44 expression. These changes collectively increase Nqo1 expression and lead to reduced hepatic oxidative stress and inflammation, potentially reversing obesity-induced TSH elevation. ↓ indicates a decrease, ↑ indicates an increase, ↔ indicates no change, and ? indicates unknown mechanisms.

This study provides novel insights into the role of RHAMM in modulating thyroid function, hepatic oxidative stress, and inflammation in obesity. Our findings support a working model where deletion of RHAMM decreases AKT/ERK signaling, which promotes the downstream GSK3 activity, leading to Nrf2 degradation. On the other hand, RHAMM deletion promotes CD44 expression, which conversely increases Nrf2 expression. Therefore, the net effect on Nrf2 expression is negligible. However, deletion of RHAMM increases Nqo1 expression possibly due to unknown direct or indirect effects from CD44. Increased Nqo1 expression leads to reduced hepatic oxidative stress and inflammation, associated with decreased TSH levels. Increased Nqo1 expression also inhibits CD44 expression, which provides a negative feedback regulation (Figure 6). This study underscores a critical role of RHAMM in thyroid dysregulation under metabolic stress of obesity. It should be highlighted that the conclusion of this study was supported by evidence obtained in male mice. Female mice were excluded from studies because of their high variability and the lack of a dietary response in TSH levels. However, this sex-specific difference in TSH responses is interesting and merits further investigations.

Our data revealed a compensatory increase in CD44 expression in the liver following *Hmmr* deletion, suggesting that RHAMM and CD44 may exhibit functional redundancy in certain biological contexts, such as inflammation and tissue remodeling [28]. Previous study has reported increased CD44 expression in the liver of obese mice, with CD44 deletion shown to reduce liver steatosis [29]. However, our study revealed no significant changes in hepatic triglyceride levels by RHAMM deletion. While hepatic triglyceride deposition remained unchanged in HFD-fed *Hmmr*^−/−^ mice, it is possible that other lipid species, such as cholesterol, may be impacted. Regardless, our triglyceride results suggest that RHAMM and CD44 may differentially regulate hepatic lipid metabolism in obesity.

We observed that RHAMM deletion in mice significantly reduced phosphorylation of AKT, ERK, and GSK3, regardless of diet. These signaling molecules play pivotal roles in cell survival, proliferation, and metabolism, and their dysregulation has been linked to hepatic steatosis, insulin resistance, and chronic inflammation in obesity [30]. In line with our findings, AKT and ERK have been previously shown to be downstream molecules of RHAMM in cancer cells [31]. GSK3 plays a central role in regulating glycogen synthesis and glucose metabolism, and its phosphorylation status is associated with insulin sensitivity [32]. Our findings of reduced GSK3 phosphorylation in *Hmmr*^−/−^ mice are novel and imply that RHAMM deletion might improve hepatic glucose handling and insulin sensitivity. Furthermore, activation of AKT and ERK pathways has been associated with hepatic inflammatory responses and oxidative damage [33], which aligns with the decreased oxidative stress and inflammation observed in the *Hmmr*^−/−^ mice.

It has been previously shown that the inhibition of GSK3 enhances Nrf2 protein stability, nuclear localization, and target gene transcription in pancreatic β cells [34]. Moreover, in bovine articular chondrocytes, HA increased Nrf2 protein expression by activating AKT, and inhibition of AKT activity or suppression of HA receptors CD44 and/or RHAMM with siRNAs prevented HA-mediated Nrf2 accumulation [19]. These results contrast with our findings, where deletion of RHAMM and decreased phosphorylation of AKT and GSK3, an indicator of AKT inactivation and GSK3 activation, led to increased transcription of the Nrf2 target gene Nqo1 in the liver of RHAMM-deficient mice. Taken together, our data suggest that RHAMM may regulate Nqo1 independently of AKT, GSK3, and Nrf2 in the liver, possibly from the compensatory upregulation of CD44. Notably, overexpression of CD44 has been shown to increase the levels of Nrf2 in human breast cancer stem-cell-like cells; the Nrf2 levels were further increased by HA treatment, whereas CD44 depletion had the opposite effect [35].

Elevated Nqo1 expression in *Hmmr*^−/−^ mice on HFD likely contributed to reduced hepatic oxidative stress. Hepatic oxidative stress not only disrupts systemic redox balance but also impairs thyroid hormone homeostasis by influencing deiodinase activity. Deiodinases are selenoenzymes highly sensitive to oxidative modifications, and their vulnerability to redox imbalance links hepatic oxidative injury to altered circulating levels of thyroid hormones and TSH [36]. The bioavailability of thyroid hormones relies heavily on peripheral conversion, particularly in the liver, which expresses Type I iodothyronine deiodinase (DIO1, which converts T4 to T3) and Type III iodothyronine deiodinase (DIO3, which inactivates T4 and T3) [36,37]. Oxidative stress and ROS, such as H_2_O_2_, can oxidatively damage the selenocysteine active site of DIO1, suppressing DIO1 gene expression and enzymatic activity [37,38], thus reducing T3 synthesis [39], a pattern consistent with subclinical or compensatory hypothyroidism.

While our study demonstrated a critical role of RHAMM in regulating thyroid function, primarily focusing on hepatic oxidative stress and inflammation, the exact role of RHAMM, especially in the context of Nrf2 antioxidant response in thyroid tissue is unknown. It has been reported that Keap1/Nrf2 signaling regulates the antioxidant defense of follicular cells, as well as iodination of thyroglobulin [40]. Specifically, Nrf2 protects the thyroid from lipid and protein oxidation induced by iodine overload [41]. Intriguingly, constitutive activation of Nrf2 by knockdown of Keap1 leads to diffuse goiter with increased size of thyroid follicles and absence of thyroid nodules or hyperplasia [42], suggesting that an appropriate level of Nrf2 is required to maintain normal thyroid function and structure. Previous study showed that mice lacking Nrf2 in the thyroid displayed normal plasma TSH or thyroid hormone levels [41], while mice lacking Keap1 had decreased T4 levels with no difference in TSH levels in early adult life, that were apparently compensated by increased TSH levels at an older age [42]. These results contrast our findings where we showed that the activation of Nrf2 by Keap1 deletion decreased plasma TSH levels. A recent transcriptomic analysis has identified gene–environment interactions between the genetic status of Keap1/Nrf2 and the dietary iodide intake [43], suggesting that differences in iodide in the diet could explain this apparent discrepancy. Regardless, it is evident that Nrf2 is an important mediator of thyroid hormone metabolism and thyroid function, either directly affecting the thyroid tissue or indirectly affecting other organs, including the liver. The characterization of the role of Nrf2 in thyroidal regulation in the context of HFD warrants further investigations. Furthermore, given the well-established role of RHAMM in ECM remodeling and cell motility, it is plausible that RHAMM may influence thyroid tissue structure and microvasculature. Future studies should include histopathological evaluation of the thyroid gland, assessing features such as follicular architecture, colloid content, inflammatory infiltration, and angiovascular remodeling [44]. These investigations will reveal novel ECM-related mechanisms of thyroid dysfunction in obesity.

From a clinical perspective, it remains unclear whether the observed elevation in plasma TSH levels in obese mice arises from primary thyroid gland dysfunction, central hypothalamic–pituitary dysregulation, or peripheral alterations in thyroid hormone metabolism. Clarifying these potential mechanisms is essential for interpreting the translational relevance. Given the classical negative feedback loop within the hypothalamic–pituitary axis, circulating T3 and T4 exert an inhibitory effect on TSH secretion [45]. Thus, plasma TSH is a highly sensitive indicator of thyroid function. TSH measurement emerges as an important means of screening patients for thyroid dysfunction, especially for ambulatory patients without other serious illnesses [46]. However, without direct measurements of free T3 and T4, the human physiological relevance of subclinical or overt hypothyroidism is uncertain and represents a limitation of our study. Moreover, the model employed in this study that focuses on obesity-induced thyroid dysfunction is unlikely to involve characteristic features of autoimmune thyroiditis. Hashimoto’s thyroiditis is the most common cause of hypothyroidism in humans and is characterized by lymphocytic infiltration and circulating thyroid autoantibodies. It is unlikely that autoimmune mechanisms are contributing to the phenotype observed in RHAMM-deficient mice. Therefore, our findings are most relevant to obesity-associated subclinical hypothyroidism, rather than autoimmune thyroid disease.

In our study, hepatic IL-6 mRNA levels were decreased by HFD feeding in both RHAMM-deficient mice and their wildtype controls to a similar extent. Although IL-6 is commonly viewed as pro-inflammatory, several studies indicate it can also play protective roles in metabolic and liver diseases. For example, IL-6 deficiency aggravates obesity-related insulin resistance and liver injury [47,48,49]. This suggests that reduced IL-6 in our model may reflect an adaptive rather than solely pro-inflammatory response. Regardless, it is important to point out that mRNA data of IL-6 were variable, possibly due to its low gene expression level. Therefore, the physiological relevance is unknown and protein expression of IL-6 will permit further clarification of the relevance of IL-6 in the context of RHAMM and thyroid regulation.

In conclusion, our findings indicate that RHAMM deletion attenuates obesity-induced TSH elevation, reduces hepatic oxidative stress, and modulates key metabolic signaling pathways in male mice. RHAMM appears to play a central role in obesity-induced endocrine and metabolic dysfunction, potentially by modulating antioxidant defense via Nqo1. These insights suggest that targeting RHAMM may offer novel therapeutic avenues for mitigating obesity-related metabolic diseases, particularly those involving thyroid dysfunction and liver inflammation.

## Data Availability

The raw data supporting the conclusions of this article will be made available by the authors on request.
